# Auditory performance in a group of elderly patients after cochlear implantation

**DOI:** 10.1007/s00405-020-06566-8

**Published:** 2021-01-11

**Authors:** Alexandros Giourgas, Martin Durisin, Anke Lesinski-Schiedat, Angelika Illg, Thomas Lenarz

**Affiliations:** Department of Otorhinolaryngology, Hannover Medical University, Karl-Wiechert-Allee 3, 30625 Hannover, Germany

**Keywords:** Cochlear implantation, Elderly, Speech perception, Ageing

## Abstract

**Purpose:**

The retrospective case review investigated the effect of cochlear implantation in subjects aged 61 years or older with respect to their auditory performance. The study also analysed the effect of age on the performance, and it drew a comparison between the outcomes of older and younger adults.

**Methods:**

The outcome in a group of 446 patients aged 61 to 89 years at the time of unilateral cochlear implantation was compared with the outcome in a group of 110 patients aged 17 to 42 years. Auditory performance was measured with open-set monosyllabic word testing and sentences in quiet and in noise.

**Results:**

In the monosyllabic word recognition test, the group of older adults performed significantly better after cochlear implantation compared with their scores prior to implantation (*p* < 0.001; *r* = 0.59). Their auditory performance correlated negatively with their age. However, the correlation was of small strength. Significant differences in auditory performance were detected between sexagenarians and octogenarians (*p* < 0.001; *r* = 0.27). Additionally, a statistically significant difference was revealed between the groups of older and younger adults in the monosyllabic word test (*p* = 0.001; *r* = 0.15).

**Conclusion:**

Elderly cochlear implant recipients can benefit significantly from cochlear implantation. Although higher age correlates negatively with auditory performance, its influence in the presented sample is small.

## Introduction

For the past decades, the age distribution in Germany’s population dramatically changed. Back in 1970, 30.0% of the German population were under 20 years of age, whereas only 2.0% were aged 80 years or older. In the year 2017, the proportion of the under-20-year-olds amounted to 18.4%, whereas 6.2% of the population were 80 years and even older [1].

In the years 2008 and 2009, a survey in Germany assessed that 31.8% of the over 65-year-olds declared to have moderate difficulties, and 6.1% even stated to encounter severe difficulties with a hearing impairment (61.1% had no difficulties). It is even assumed that the prevalence for a hearing impairment is underrated [2].

In case of difficulties with a hearing impairment due to severe to profound sensorineural hearing loss, cochlear implantation can be considered as the mainstay of hearing rehabilitation. A cochlear implant is an electronic prosthesis that stimulates the cochlear nerve. Its benefits in terms of environmental sound awareness and speech perception are well documented.

Both the mentioned prevalence of a hearing impairment and the tendency in the population’s age distribution are reflected in the demographics of the patients who were treated in the Department of Otorhinolaryngology of the clinic. Specifically, the percentage of the patients who received a cochlear implant at an age of 65 years or older grew over the years from 6.4% in 1990 to 20.2% in 2017. In this context, two of the questions to be addressed are whether elderly patients do gain benefit from cochlear implantation, and whether a higher age at implantation does have negative impact on the patient’s post-operative auditory performance.

Numerous previous studies have examined the auditory performance of elderly patients after cochlear implantation ([3–9], compare the synopsis from Clark et al. [10]). Friedland et al. [5] state that despite the importance of each study, resilient conclusions about the effect of age may commonly be held up by small cohorts, broad inclusion criteria (which primarily affects comparison groups), or methodical differences.

The aim of this retrospective case review was to assess the auditory performance of elderly patients before and after cochlear implantation, to investigate the effect of age on the performance, and to draw a comparison between the elderly and a population of significantly younger adults. Due to its large, relatively homogenous (i.e. unilaterally implanted; postlingually deafened) and, in terms of age, narrowly specified cohorts, the present analysis shall provide additional insight to a topic of increasing importance, thus supporting generalizable statements about the efficacy of cochlear implantation in a specific patient group.

## Materials and methods

The present study focusses on elderly and geriatric patients who received a cochlear implant and compares them to cochlear implant recipients of younger age. All included patients underwent the same preoperative medical, audiologic, radiologic, and pedagogic evaluation in order to determine indication and suitability for cochlear implantation. Currently, the audiological criteria are under revision [11]. According to the recommendations of the German Society of Oto-Rhino-Laryngology, Head and Neck Surgery, from an audiological point of view, a CI is indicated when the discrimination of monosyllabic words is equal or less than 60% under aided condition and 65 dB sound pressure level [12]. All patients were implanted unilaterally with a Cochlear Nucleus (M-24, RE-24, CI512, CI532, CI422), Advanced Bionics (HiRes90k), or Med-El (Pulsar, Concerto, Sonata, Synchrony) device. Patients who underwent reimplantation during the observation period, and patients with light to moderate hearing loss on their contralateral ear, as well as unilaterally deaf patients, were excluded from the study. Patients without intelligible connected speech (i.e. prelingually deafened) were also excluded from the study. All patients received technical fitting and hearing training from a specialized team under standardized conditions during a 5-day first fitting period and were monitored in the context of aftercare.

A retrospective data analysis was performed using the audiologic and medical records of all subjects. Testing was administered on one ear solely using standardized open-set speech perception testing prior to implantation (aided with conventional amplification whenever feasible), and at the 12-month-appointment after the first fitting of the cochlear implant. Auditory performance was measured in free field via loudspeaker with the Freiburger Monosyllables Test (FMT) [13] at a presentation level of 65 dB SPL and the Hochmair-Desoyer/Schulz/Moser sentence test in noise (HSMn) [14] at a 65 dB SPL/10 dB signal-to-noise ratio.

Mean (M) and median (Md) scores (per cent correct) of all auditory outcomes were calculated. Since the assumptions of parametric statistical techniques (i.e. assumption of normality and/or homogeneity of variance) were mostly not met, analyses were performed by using nonparametric techniques. The Mann–Whitney *U* test was administered to test for differences between two independent groups on a continuous measure. Differences between three or more groups were analysed using the Kruskal–Wallis test. A Wilcoxon signed rank test was used for repeated measures (e.g. pre–post comparison). A Friedman test was performed to address the question of whether there is a change in auditory performance across four points in time in each group. Finally, the Spearman’s rank order correlation was used to determine relationships among two continuous variables. Strength of relationships as well as effect sizes (*r* = *z*/square root of *n*) was interpreted using Cohen’s [15] criteria. Statistical analyses were performed using IBM SPSS Statistics 26.

## Results

A total of 556 adults who received a cochlear implant were included in the study. A total of 446 patients who received their cochlear implant at an age between 61 and 89 years formed Group 1. In order to compare the cohort of elderly patients with younger cochlear implant recipients, clinical and audiologic records of 110 randomly selected deafened adults were assigned to Group 2. Exclusion criteria and implantation period were set according to Group 1. A database query extracted the identification numbers (ID) of all patients who met the criteria. The final sample of cases was randomly extracted from the pool of IDs by use of the according function in IBM SPSS. Data were revised in order to avoid misassignments, i.e. due to misentries in the database. The age at implantation in Group 2 ranged from 17 to 42 years, assuring its mean age being significantly lower (*z* =  − 16.256, *p* < 0.001). There was no significant difference between Group 1 (Md = 23, *n* = 430) and Group 2 (Md = 21.5, *n* = 108) related to their duration of hearing loss (*z* =  − 1.358, *p* = 0,175). Further demographics of Group 1 and Group 2 are listed in Table 1.

**Table 1:** Demographic information summarizing the study population

	Group 1 [*n* = 446]	Group 2 [*n* = 110]
Gender		
Female	226 (50.7%)	65 (59.1%)
Male	220 (49.3%)	45 (40.9%)
Age at implantation [years]		
Mean (SD)/median (range)	72.9 (6.3)/72.8 (61.1–89.5)	32.0 (7.5)/33.4 (17.2–42.4)
Duration of hearing loss [years] Mean (SD)/Median (range)	27.2 (19.9)/23.0 (0.0–82.0)	22.0 (11.6)/21.5 (0.0–42.0)
Implantation period	14.01.2004 to 14.10.2016	11.09.2003 to 01.04.2016
Type of implant		
Advanced bionics MedEl Nucleus	141 (31.6%) 87 (19.5%) 218 (48.9%)	31 (28.2%) 13 (11.8%) 66 (60.0%)
Side of implantation Right/left	237 (53.1%)/209 (46.9%)	55 (50.0%)/50 (50.0%)
Aetiology Genetical Iatrogenic Infection Neural Specific inner ear disease Trauma exposure Unknown	*n* (%) 12 (2.7) 4 (0.9) 37 (7.3) 4 (0.9) 42 (9.4) 19 (4.3) 328 (73.5)	*n* (%) 5 (4.5) 0 (0.0) 24 (21.8) 1 (0.9) 9 (8.2) 2 (1.8) 64 (58.2)
Comorbidities		
One Multiple Cognitive/neurological None Unknown	125 (28.0) 23 (5.2) 21 (4.7) 274 (61.4) 3 (0.7)	20 (18.2) 2 (1.8) 3 (2.7) 85 (77.3) 0 (0.0)

Table 2 contains an overview of the pre- and post-operative FMT scores in Group 1 and Group 2. A Mann–Whitney *U* test indicated no significant difference in preoperative FMT scores between both groups (*z* =  − 1.219, *p* = 0.223).

**Table 2:** Pre- and post-operative Freiburger Monosyllables Test (FMT) scores [% correct] for Group 1 and Group 2

	FMT score pre-implantation		FMT 12 months post-activation	
	Group 1	Group 2	Group 1	Group 2
*N* Mean (SD) Median (range)	441 6.6 (12.3) 0.0 (0–55.0)	103 5.0 (10.6) 0.0 (0–45.0)	395 52.2 (25.7) 55.0 (0–100)	105 61.1 (27.7) 70.0 (0–95.0)

The median score in Group 1 improved from Md = 0.0% (*n* = 441) prior to cochlear implantation to Md = 55.0% (*n* = 395) at the 12-month-appointment. A Wilcoxon signed rank test was conducted to compare FMT scores in Group 1 before and after implantation. There was a statistically significant improvement in FMT score (*z* =  − 16.43, *p* < 0.001) with a large effect size (*r* = 0.59).

A Spearman ranked order correlation indicates no relationship between the elderly patients’ FMT scores prior to and 12 months after cochlear implantation (*r* = 0.075, *n* = 391, *p* = 0.137).

**Fig. 1:**
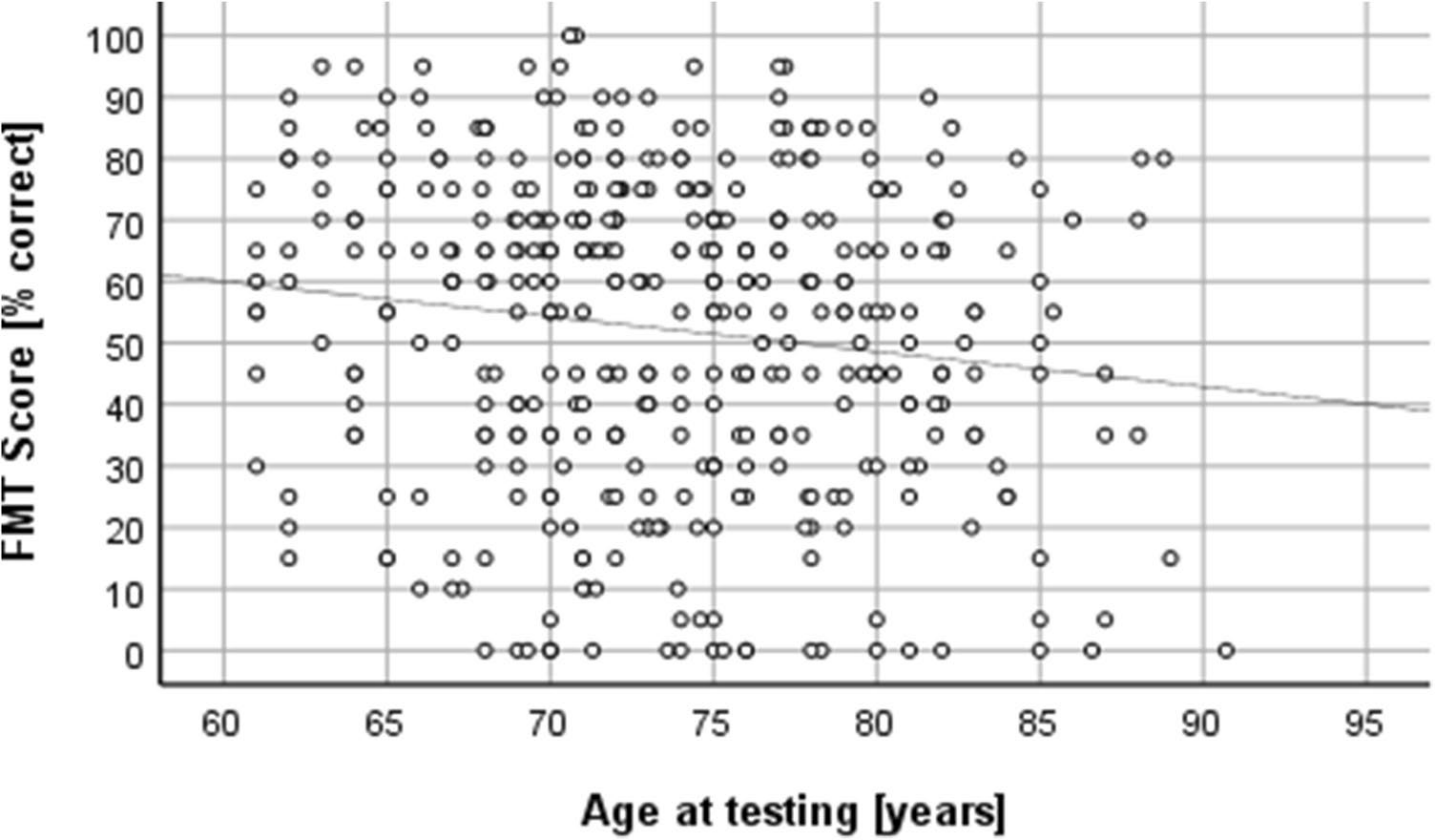
Correlation between the scores in the Freiburger Monosyllables Test (FMT) and the age at testing for Group 1

**Fig. 2:**
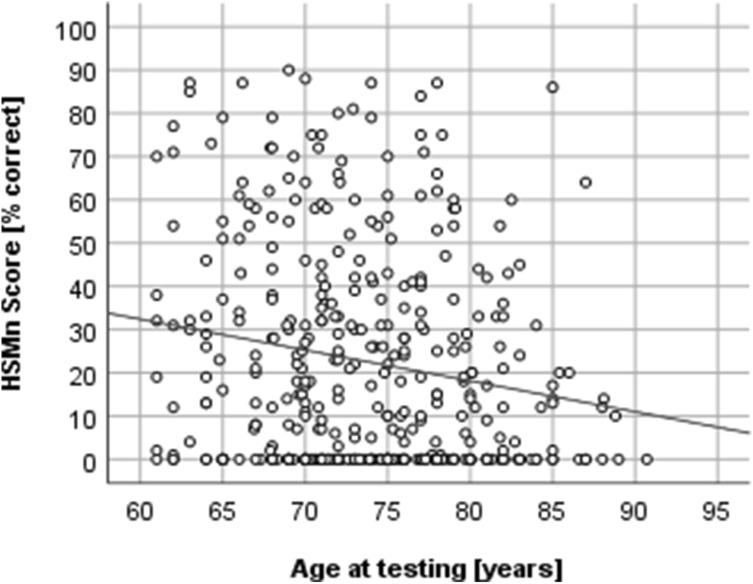
Correlation between the scores in the Hochmair-Desoyer/Schulz/Moser-Sentence test in noise (HSMn) and the age at testing for Group 1

Analysis of the correlations between the age at the 12-month-assessment and the speech perception scores via the Spearman ranked order correlation clarifies that there is a negative relationship between both variables in Group 1. In other words, higher age is associated with lower speech perception scores (FMT: *r* =  − 0.127, *n* = 395, *p* = 0.012; HSMn: *r* =  − 0.162, *n* = 386, *p* = 0.001). According to Cohen’s guidelines, both correlations are of small strength. The scatter plots illustrate the relationship (Figs. 1, 2).

In Group 2, there is minimal to none relationship between age and speech perception scores 12 months after cochlear implantation (FMT: *r* =  − 0.063, *n* = 105, *p* = 0.525; HSMn: *r* =  − 0.061, *n* = 102, *p* = 0.544).

A Mann–Whitney *U* test yielded a significant difference in the auditory performance across Group 1 and Group 2 for the FMT (*p* = 0.001; *z* =  − 3.445; *r* = 0.15). On the contrary, in the HSMn, no significant difference was found between both groups (*p* = 0.222; *z* =  − 1.222; *r* = 0.06).

For the purpose of further analyses, Group 1 was split up into three subgroups according to their age at the 12-month-assessment. Group 1a contained 108 patients aged 61.0 to 69.8 years (M = 66.2), Group 1b contained 222 patients aged 70.0 to 79.8 years (M = 74.3), and Group 1c 68 contained patients aged 80 to 90.7 years (M = 83.1). Table 3 gives an overview of the speech perception scores in each subgroup (1a, 1b, 1c). Additionally, the results of Group 2 are displayed.

**Table 3:** Scores [% correct] in the Freiburger Monosyllables Test (FMT) and the Hochmair-Desoyer/Schulz/Moser-Sentence test in noise (HSMn) for the subgroups 1a, 1b, 1c, and for Group 2

	Mean age at testing (range)	FMT 12 months post-activation mean (SD)/median (range) [*n*]	HSMn 12 months post-activation mean (SD)/median (range) [*n*]
Group 1	73.6 (61.0–90.7)	52.2 (25.7)/55.5 (0–100) [395]	22.7 (24.8)/15.0 (0–90) [386]
Group 1a	66.2 (61.0–69.8)	57.2 (24.6)/65.0 (0–95) [107]	28.7 (26.9)/23.5 (0–90) [104]
Group 1b	74.3 (70.0–79.8)	51.9 (26.0)/55.0 (0–100) [221]	22.2 (24.6)/14.0 (0–88) [217]
Group 1c	83.1 (80.0–90.7)	45.3 (24.9)/45.0 (0–90) [67]	14.5 (18.9)/9.0 (0–86) [65]
Group 2	33.0 (18.3–43.5)	61.0 (27.7)/70.0 (0–95) [105]	26.8 (27.9)/17.0 (0–91) [102]

A Kruskal–Wallis test reveals a significant difference across the three subgroups 1a, 1b, 1c in the FMT results 12 months post-activation of the device (*p* = 0.011) as also in the HSMn test results (*p* = 0.002). Follow-up Mann–Whitney *U* tests between pairs of the subgroups necessitates an adjustment of the alpha value (corrected alpha value following Bonferroni = 0.05/3 = 0.017). After Bonferroni adjustment, no significant differences were found between the subgroups 1a and 1b (FMT: *p* = 0.081; HSMn: *p* = 0.025) and the subgroups 1b and 1c (FMT: *p* = 0.067; HSMn: *p* = 0.060). However, significant differences were found between the subgroups 1a and 1c (FMT: *p* = 0.002; *z* =  − 3.039; *r* = 0.230; HSMn: *p* < 0.001; *z* =  − 3.482; *r* = 0.268).

**Fig. 3:**
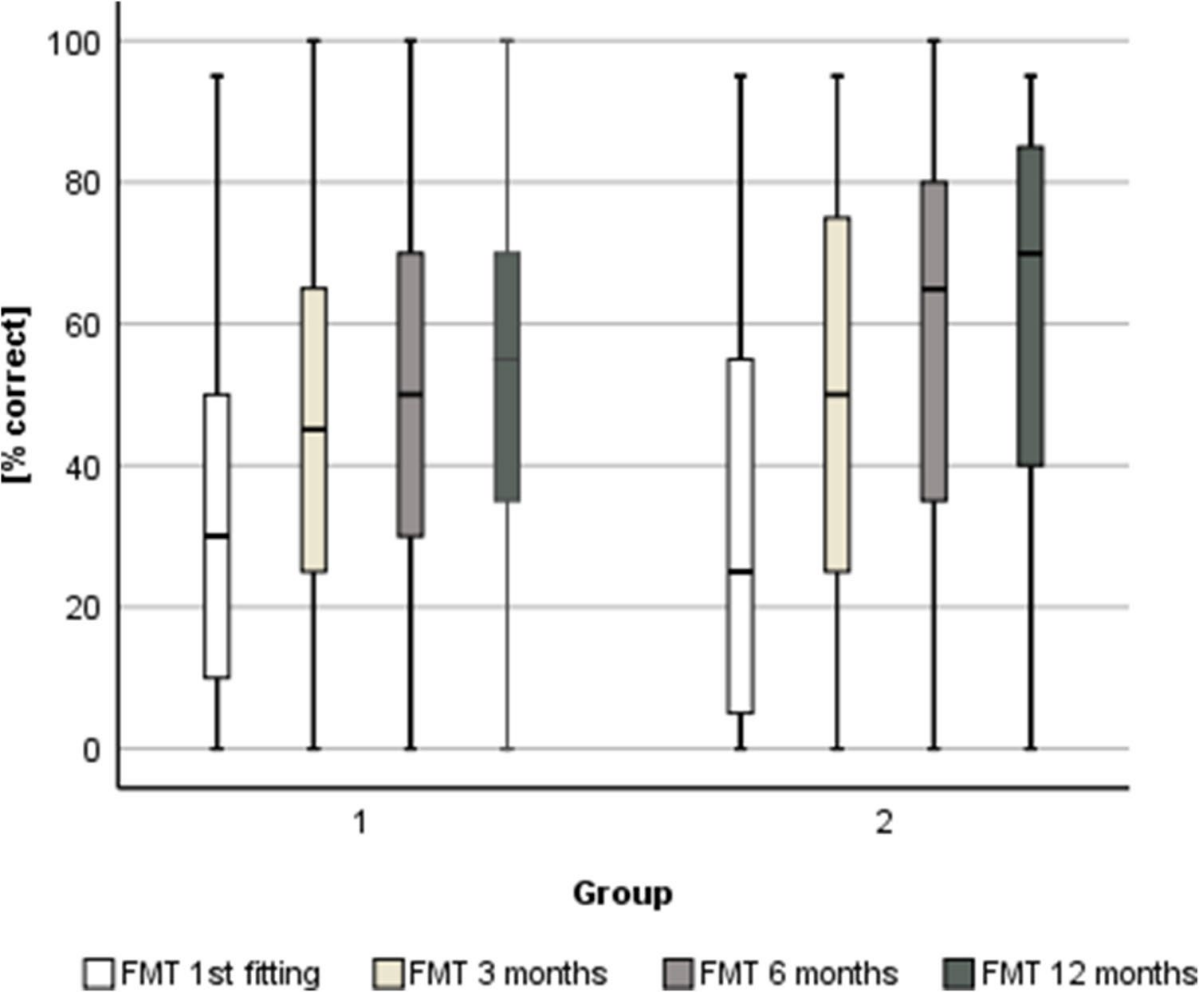
Scores [% correct] in the Freiburger Monosyllables Test (FMT) for Group 1 and 2 depending on the time at evaluation (i.e. first fitting [Group 1 *n* = 445; Group 2 *n* = 110]; 3 months [Group 1 *n* = 415; Group 2 *n* = 103]; 6 months [Group 1 *n* = 406; Group 2 *n* = 93]; 12 months [Group 1 *n* = 395; Group 2 *n* = 105] post-implantation)

**Fig. 4:**
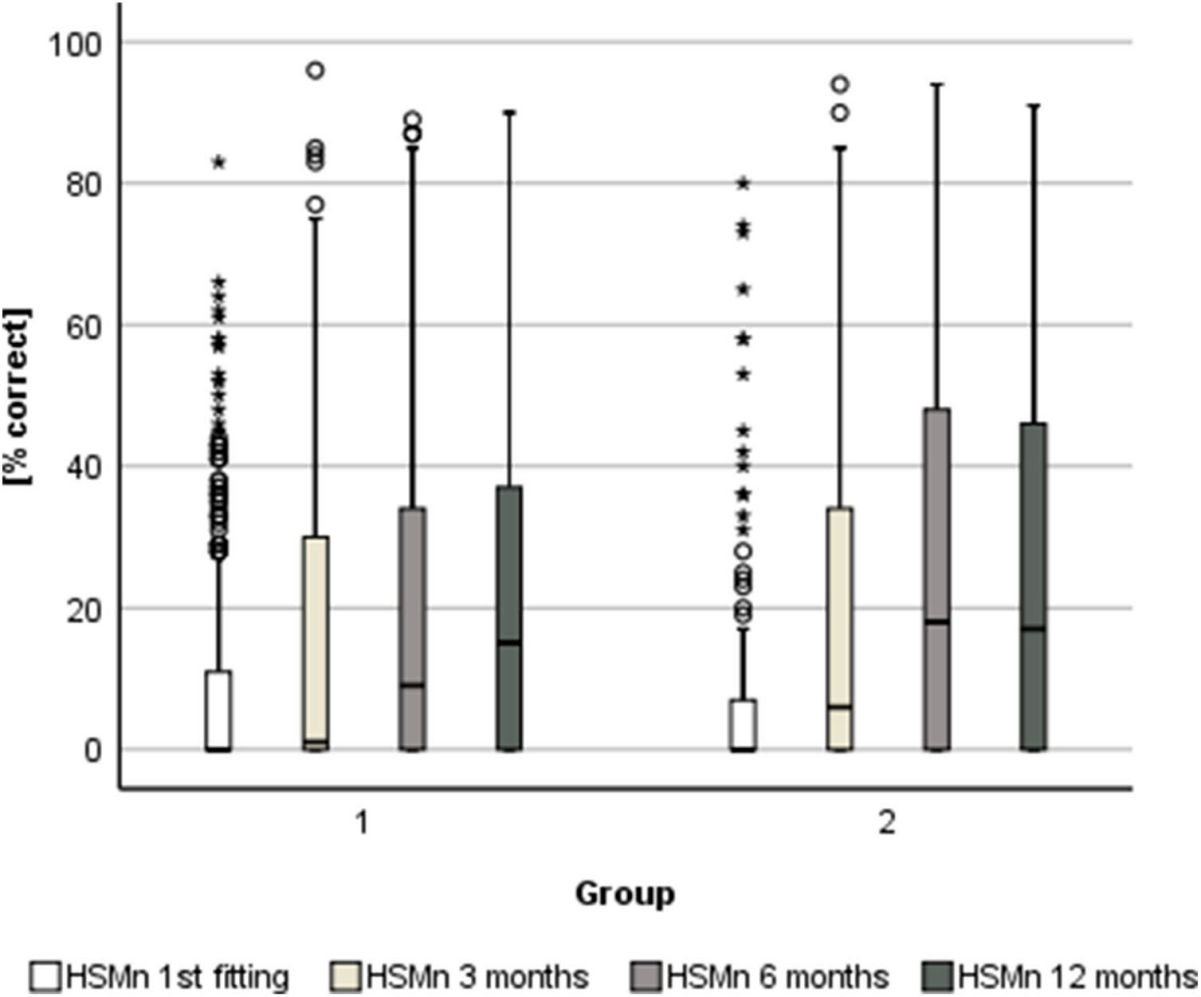
Scores [% correct] in the Hochmair-Desoyer/Schulz/Moser-Sentence test in noise (HSMn) for Group 1 and 2 depending on the time at evaluation (i.e. first fitting [Group 1 *n* = 440; Group 2 *n* = 110]; 3 months [Group 1 *n* = 409; Group 2 *n* = 101]; 6 months [Group 1 *n* = 400; Group 2 *n* = 91]; 12 months [Group 1 *n* = 386; Group 2 *n* = 102] post-implantation)

Figures 3 and 4 depict additional intermediate results in both groups (first fitting, 3- and 6-month follow-up).

Results of a Friedman test suggest that there is a significant difference in the scores across the four time points in both groups (Group 1: chi-square (3, *n* = 331) = 200.01, *p* < 0.05; Group 2: chi-square (3, *n* = 81) = 72.14, *p* < 0.05).

The duration of hearing loss does not seem to correlate with the auditory performance in Group 1 (FMT: *r* =  − 0.076, *n* = 381, *p* = 0.138; HSMn: *r* =  − 0.007; *n* = 373; *p* = 0.895). Furthermore, gender does not have significant impact on auditory performance in Group 1 (FMT: *p* = 0.732; HSMn: *p* = 0.879).

In Group 1, comorbidities were documented in 37.9% of the subjects. Several patients were afflicted with more than one comorbidity (i.e. heart insufficiency as well as limited motor skills). The comorbidities were categorized accordingly, with cognitive and/or neurological diagnoses forming a separate category, comprising 21 subjects (i.e. dyslexia in one case; condition after apoplectic insult in nine cases; suspected dementia or concentration and memory issues in eight cases). Table 4 summarizes the speech perception scores in Group 1 depending on the above-mentioned categories. However, statistical comparisons were not performed due to the small samples.

**Table 4:** Scores [% correct] in the Freiburger Monosyllables Test (FMT) and Hochmair-Desoyer/Schulz/Moser-Sentence test in noise (HSMn) for Group 1 depending on comorbidities

	FMT preoperatively	FMT 12 months post-activation	HSMn 12 months post
Category	Mean (SD)/median (range) [*n*]	Mean (SD)/median (range) [*n*]	Mean (SD)/median (range) [*n*]
One comorbidity Multiple comorbidities Cognitive/neurological None	6.1 (12.3)/0.0 (0–55) [123] 4.3 (11.4)/0.0 (0–40) [22] 2.3 (4.7)/0.0 (0–15) [20] 7.9 (13.6)/0.00 (0–60) [273]	48.1 (25.7)/52.5 (0–95) [110] 51.7 (29.9)/60.0 (0–85) [21] 46.3 (26.9)/50 (0–90) [14] 54.7 (24.9)/60.0 (0–100) [248]	23.0 (25.9)/16.5 (0–88) [106] 20.1 (23.2)/12.5 (0–79) [20] 15.0 (17.5)/3.0 (0–46) [14] 23.3 (24.8)/15.0 (0–90) [244]

## Discussion

The cochlear implant has been labelled as the most successful and effective implantable neural prosthesis in terms of restoring sensitive functions to its recipients [16]. Previous studies addressed the question, whether this applies as well to elderly and/or geriatric candidates. The present analysis investigated 446 elderly patients and compared their auditory performance with a control sample of 110 younger adults. The study revealed a significantly increased speech perception in the elderly 12 months after cochlear implantation compared with their preoperative status. This stands in line with similar studies [3–5, 7–9, 17–21].

The overall results suggest that age has a negative impact on auditory performance after cochlear implantation. However, the strength of the correlation is small. A comparison between the group of elderly and younger subjects revealed a statistically significant effect of age on the understanding of monosyllabic words.

According to comparable publications [4, 5, 19, 22], the auditory performance tendentially decreases with growing age. Orabi et al. [23] and Lin et al. [6] publicized analogical results regarding the auditory performance in noise. Lin et al. [6] calculated that for every increasing year of age with cochlear implant, the scores lessened by 1.3 percentage points. Similarly, Leung [22] reckoned a (non-significant) 0.005% detriment in consonant nucleus-consonant word score (CNC) in 258 patients for each year older than 65 years. (In the present study, a linear correlation was not performed due to the violation of necessary assumptions about the data).

Friedland and colleagues [5] detected a higher score distribution in the elderly in the Hearing in Noise Test-Noise. However, a higher score distribution in the elderly could not be observed in the present study (see Table 3: HSMn: Group 1 SD = 24.8; Group 2 SD = 27.9). Furthermore, the group of elderly did not differ significantly in the HSMn scores in comparison with the group of younger patients. This result may be surprising, particularly because the FET scores speak for a significantly lower auditory discrimination in Group 1. The result may arise from the applied method; the HSMn test presents meaningful sentences in a stationary broadband noise. Under such condition, and despite the given pressure, subjects might still be able to compensate the reduced auditory discrimination by use of their semantic memory. According to Meister et al. [24], modulated maskers might differentiate better between groups. This aspect should be considered in future studies.

An in-group comparison within the elderly showed a statistically significant difference between subjects being in their 60 s (Group 1a, mean age = 66.2) and their 80 s (Group 1c, mean age = 83.1) in both, FMT and HSMn. With every decade of age growing, the median FMT score decreased by 10% points, and the mean HSMn score by 8.5 and 7.7% points, respectively. This finding corresponds with results of Roberts et al. [25], where octogenarians scored poorer in the consonant-nucleus-consonant (CNC) testing compared with patients 60 to 69 years of age.

According to Leung [22], the design of the study regarding the set cut-off-age influences the conclusions. Other than in most similar studies, no certain cut-off-age was set in the present evaluation when comparing groups with each other (i.e. 60 years: [6, 26]; 65 years: [3, 21, 22, 25, 27–29]; 70 years: [4, 19, 30]; 75 years: [31]; 80 years: [32]). In particular, the group of elderly patients (Group 1) was compared with a control group of considerably younger patients (Group 2). The mean chronological age at implantation in Group 1 was significantly higher than that in the 110 candidates of Group 2, with the oldest patient of Group 2 being 42.4 years of age (thus 18.7 years younger than the youngest patient in Group 1). In contrast to other investigations of comparable design [3, 8, 19, 28, 32], a between-groups-comparison revealed significant differences in the monosyllabic word recognition.

Further studies revealed, at least partially, significant differences between groups of younger and older CI recipients. Chatelin and colleagues [4] set the cut-off age at 70 years and found a statistically significant rate of change in the monosyllabic word recognition test (CNC) with the younger patients outperforming the elderly. Similarly, Vermeire et al. [17] discovered a significant difference in the audiologic performance between a group of younger patients and the oldest subgroup of their geriatric population. Lundin et al. [7] located a significantly higher post-operative monosyllabic word comprehension in younger patients compared with patients 79 years or older. Carlson et al. [32], as a partial result, found significant disadvantages in patients older than 80 years in the post-operative AzBio sentence scores.

Numerous factors may affect speech perception scores after cochlear implantation. Findings of earlier studies (see [5, 6]) support the assumption that a good preoperative speech perception influences the performance after cochlear implantation positively. However, the strength of the correlation in the present study was vanishingly small (*r* = 0.075). The duration of hearing loss has emerged as a critical parameter in determining speech recognition capability in quiet conditions [33]. According to Hirschfelder, a longer duration of deafness was generally correlated with lower monosyllabic word scores [34]. In contrast, the present data indicate a very small negative correlation between the duration of profound deafness and the auditory performance post CI.

Herzog et al. [27] underlined that their group of elderly CI recipients needed significantly longer to catch up with the scores of younger candidates after cochlear implantation in the HSM test within an observation period of 6 years. Results of the present analysis suggest that the HSM scores in the group of younger patients (Group 2) stabilize after 6 months whereas Group 1 catches up in the 12-month-appointment (Fig. 3). According to the Friedman test, the auditory performance improves significantly within the first 12 months after first fitting. In this context, it should be considered that all included candidates were supported by specialized staff in context of the first fitting period and the regular aftercare in the Department for Otorhinolaryngology. Additionally, whenever deemed necessary, outpatient auditory therapy is being prescribed, mostly within the first 12 months after implantation.

Elderly patients often suffer from comorbidities that could cause medical complications and lessen audiologic success. Following the present results, except for neurological comorbidities (i.e. condition after apoplectic insult), additional handicaps do not seem to influence the speech comprehension negatively. However, due to the small subgroup sizes, tests of significance were not performed. Additionally, the reservation has to be made that tests of the mental functions were yet to be systematically conducted with the included candidates—a factor that further ongoing studies in our department will be examining.

An important aspect of the cochlear implantation in elderly patients is the question of possible higher risk of complications during anaesthesia. In this aspect, the influence of anaesthesia on mortality and postoperative morbidity is still controversially discussed in the medical literature [35–37]. The incidence of true anaesthesia-related mortality is low (0.014–0.16%) [38–40]. The rate of early and late complications depends on the study and varies between 6.9 and 25% [41–44]. A relevant predictor to evaluate the risk of general anaesthesia represents the classification of American Society of Anaesthesiologists (ASA score) [45]. It must be emphasized that age alone does not reflect the health status of the patient. Comorbidities play an important role to estimate possible anaesthesia risks [42, 46]. In order to make the anaesthesia as safe as possible in elderly, a careful evaluation of medical history and peri- as well as post-operative management is required. Furthermore, a cochlear implantation under local anaesthesia is offered to the patient and performed in some cases.

In order to be successful postoperatively, a continuous and reliable aftercare is essential. Therefore, when considering a cochlear implantation with a patient of higher age, other factors should be taken into account, such as his overall compliance and motivation, his ability to handle the device and care for it, his social environment, cognitive skills, or his capability to attend follow-up appointments.

The present study suggests that elderly cochlear implant recipients benefit from the intervention. Albeit they achieved lower scores in the monosyllabic word test in quiet, their results in the sentence test in noise were comparable to the group of significantly younger patients. Due to the sample’s size and homogeneity, the study may represent an important contribution in understanding the effects of cochlear implantation in elderly patients. However, some reservations should be taken into account, such as a more precise investigation of the impact of comorbidities and cognitive skills in the group of elderly, an extended observation period in order to ensure performance stability, the use of tests in modulated noise for the purpose of better discrimination between the groups, or even a tighter focus on octogenarians and older.
